# Environmental prevalence of toxigenic *Vibrio cholerae* O1 in Bangladesh coincides with *V*. *cholerae* non-O1 non-O139 genetic variants which overproduce autoinducer-2

**DOI:** 10.1371/journal.pone.0254068

**Published:** 2021-07-02

**Authors:** Iftekhar Bin Naser, Tushar Ahmed Shishir, Shah Nayeem Faruque, M. Mozammel Hoque, Anamul Hasan, Shah M. Faruque

**Affiliations:** 1 School of Environment and Life Sciences, Independent University Bangladesh, Bashundhara, Dhaka, Bangladesh; 2 Department of Mathematics and Natural Sciences, BRAC University, Dhaka, Bangladesh; 3 Laboratory Sciences and Services Division, International Centre for Diarrhoeal Disease Research, Bangladesh, Dhaka, Bangladesh; 4 Department of Biochemistry and Microbiology, North South University, Dhaka, Bangladesh; Nitte University, INDIA

## Abstract

Prevalence of toxigenic *Vibrio cholerae* O1 in aquatic reservoirs in Bangladesh apparently increases coinciding with the occurrence of seasonal cholera epidemics. In between epidemics, these bacteria persist in water mostly as dormant cells, known as viable but non-culturable cells (VBNC), or conditionally viable environmental cells (CVEC), that fail to grow in routine culture. CVEC resuscitate to active cells when enriched in culture medium supplemented with quorum sensing autoinducers CAI-1 or AI-2 which are signal molecules that regulate gene expression dependent on cell density. *V*. *cholerae* O1 mutant strains with inactivated *cqsS* gene encoding the CAI-1 receptor has been shown to overproduce AI-2 that enhance CVEC resuscitation in water samples. Since *V*. *cholerae* non-O1 non-O139 (non-cholera-vibrios) are abundant in aquatic ecosystems, we identified and characterized naturally occurring variant strains of *V*. *cholerae* non-O1 non-O139 which overproduce AI-2, and monitored their co-occurrence with *V*. *cholerae* O1 in water samples. The nucleotide sequence and predicted protein products of the *cqsS* gene carried by AI-2 overproducing variant strains showed divergence from that of typical *V*. *cholerae* O1 or non-O1 strains, and their culture supernatants enhanced resuscitation of CVEC in water samples. Furthermore, prevalence of *V*. *cholerae* O1 in the aquatic environment was found to coincide with an increase in AI-2 overproducing non-O1 non-O139 strains. These results suggest a possible role of non-cholera vibrios in the environmental biology of the cholera pathogen, in which non-O1 non-O139 variant strains overproducing AI-2 presumably contribute in resuscitation of the latent pathogen, leading to seasonal cholera epidemics. **Importance**. Toxigenic *Vibrio cholerae* which causes seasonal epidemics of cholera persists in aquatic reservoirs in endemic areas. The bacteria mostly exist in a dormant state during inter-epidemic periods, but periodically resuscitate to the active form. The resuscitation is enhanced by signal molecules called autoinducers (AIs). Toxigenic *V*. *cholerae* can be recovered from water samples that normally test negative for the organism in conventional culture, by supplementing the culture medium with exogenous AIs. *V*. *cholerae* belonging to the non-O1 non-O139 serogroups which do not cause cholera are also abundant in natural waters, and they are capable of producing AIs. In this study we characterized *V*. *cholerae* non-O1 non-O139 variant strains which overproduce an autoinducer called AI-2, and found that the abundance of the cholera pathogen in aquatic reservoirs correlates with an increase in the AI-2 overproducing strains. Our results suggest a probable role of these variant strains in the environmental biology and epidemiology of toxigenic *V*. *cholerae*, and may lead to novel means for surveillance, prevention and control of cholera.

## Introduction

The natural habitat of the species *Vibrio cholerae* is the aquatic ecosystem, and comprises more than 250 O-serogroups. However, only toxigenic strains of *V*. *cholerae* O1 and rarely that of O139 cause cholera in humans and may spread as epidemics [1,2]. These strains carry a set of virulence associated genes which encode cholera toxin and a colonization factor known as toxin coregulated pilus [2]. Strains of the remaining serogroups collectively referred to as *V*. *cholerae* non-O1 non-O139 or “non-cholera” vibrios do not usually carry these major virulence genes, and do not cause cholera, but may be rarely associated with mild diarrhea or extraintestinal infections [2]. *V*. *cholerae* persists in the aquatic reservoirs, mostly in a dormant state known as viable but non-culturable (VBNC) form, alternatively referred to as conditionally viable environmental cells (CVEC) [3,4], and this dormant state is assumed to allow survival of the bacteria in adverse conditions [1,5].

In cholera-endemic regions such as Bangladesh, viable *V*. *cholerae* can be readily found in water during cholera epidemics which occur seasonally [6]. However, during non-epidemic periods, *V*. *cholerae* mostly exist as CVEC, and techniques such as fluorescent antibody based microscopy or special enrichment culture techniques [4,7] are required to detect these dormant cells. CVEC are aggregates of cells which are embedded in a matrix of Vibrio extracellular polysaccharide (VPS), and their formation involves quorum sensing, a regulatory network of gene expression that is dependent on the density of bacterial cells [8]. Quorum sensing in *V*. *cholerae* involves at least three autoinducers (CAI-1, AI-2 and DPO) and their respective receptors CqsS, LuxP, and VqmA together with intricate signal transduction cascades [9–14]. These three AIs in different combinations may allow *V*. *cholerae* to sense the total bacteria in a mixed population as well as the relative concentration of Vibrio cells present, and accordingly regulate appropriate metabolic pathways [9]. CAI-1 and AI-2 are sensed by the membrane-bound receptors CqsS and LuxPQ which channel information into a common regulatory pathway [12,13]. On the other hand, DPO is detected by the VqmA receptor-transcription factor in the cytoplasm. VqmA activates expression of *vqmR* gene that encodes the VqmR small RNA (sRNA) [10,15,16] which regulates target mRNAs.

We demonstrated recently that supplementation of culture medium with exogenous autoinducers CAI-1 and AI-2 enhances resuscitation of CVEC, and that certain variant *V*. *cholerae* O1 strains inherently overproduce AI-2 [4,17], and thus may contribute to resuscitation of the dormant cells. These AI-2 overproducing strains invariably carried mutational inactivation of *cqsS* gene which encodes the sensor for CAI-1. Furthermore, mutational analysis of the *cqsS* gene in a laboratory strain C6706 also confirmed this phenomenon [17]. However, it remains unclear whether such rare variants of *V*. *cholerae* O1 strains can be the source of sufficient levels of autoinducers to effectively resuscitate CVEC under natural conditions. Since *V*. *cholerae* non-O1 non-O139 strains or the non-cholera virbios are known to be significantly more abundant in environmental waters, we explored the possibility that a subpopulation of *V*. *cholerae* non-O1 non-O139 might also sustain mutations in the *cqsS* gene resulting in overproduction of AI-2 and thus effectively contribute towards resuscitation of dormant *V*. *cholerae* O1. Therefore, we identified and characterized non-O1 non-O139 isolates from the environment for possible overproduction of autoinducers. Selected isolates were subjected to whole genome sequencing, and analysis of relevant genes carried by the variant strains as compared to those of typical *V*. *cholerae* O1 and non-O1 strains. Subsequently, we systematically monitored possible association of these variant strains with seasonal increase in toxigenic *V*. *cholerae* O1 in the aquatic environment in Bangladesh.

## Materials and methods

### Bacterial strains, and culture condition

Bacterial strains analyzed in this study were either isolated from environmental water samples in Dhaka, or obtained from our culture collection. Relevant characteristics of various genetic constructs and control strains used in this study are presented in Table 1. Unless stated otherwise, the bacterial strains were routinely cultured in Luria-Bertini broth (LB) or LB-agar containing appropriate antibiotics at 37° C. For isolation of environmental *V*. *cholerae* strains, water samples were subjected to an enrichment culture technique referred to as AST described by us previously [7]. In brief, 5 ml of each water sample was added to 2.5 ml of 3X concentrated bile peptone medium (BP, 1% peptone, 0.5% taurocholic acid, 1% NaCl, pH 9.0), and incubated for 5h at 37°C. Dilutions of the enrichment cultures were spread on taurocholate tellurite gelatine agar (TTGA) [18] plates containing streptomycin (70 μg/ml), as well as on a set of TTGA plates devoid of the antibiotic. Suspected *V*. *cholerae* O1 colonies were picked from the antibiotic-containing plates, and subjected to standard biochemical and serological tests as described previously [7]. For non-O1 non-O139 strains, suspected colonies were picked from the plates devoid of antibiotic, and subjected to further analysis.

**Table 1:** Description of bacterial strains and plasmids included in this study.

Strain/plasmid	Relevant Genotype	Description
pJZ176	The *cqsA* gene cloned in pTAC under an IPTG inducible *lac* promoter	Recombinant plasmid carrying CAI-1 synthase gene
pJZ365	The *luxS* gene cloned in pTAC under an IPTG inducible *lac* promoter	Recombinant plasmid carrying AI-2 synthase gene
DH5α (pJZ176)	*E*. *coli* DH5α carrying pJZ176	Produces CAI -1 on IPTG induction
DH5α (pJZ365)	*E*. *coli* DH5α carrying pJZ365	Produces AI-2 on IPTG induction
DH5α (pTAC)	*E*. *coli* DH5α carrying pTAC	Carries the empty vector pTAC
C6706	*V*. *cholerae* O1 El Tor strain used to construct various deletion mutants	*V*. *cholerae* O1 El Tor biotype strain
IMGL-11	C6706, Δ*cqsA*	Deficient in CAI-1 production
EC20699	C6706,*luxS*::TnFGL3	Deficient in AI-2 production
IMGL-12	C6706,*luxS*::TnFGL3 Δ*cqsA*	Deficient in both CAI-1 and AI-2
MGL-13	C6706,luxP::TnFGL3 cqsS::cat	Deficient in both AI-1 and AI-2 sensors
86V1216 107V1216	Environmental *V*. *cholerae* non-O1 non-O139 variants carrying mutations in *CqsS* gene	*V*. *cholerae* non-O1 non-O139 strains which overproduce AI-2
126V0117	Environmental *V*. *cholerae* non-O1 non-O139 strain	Nontoxigenic environmental *V*. *cholerae*
MM920	*V*. *cholerae* Δ*cqsA*Δ*luxQ* (pBB1). Plasmid pBB1 carries the *luxCDABE* operon of *V*. *harveyi*	Produces bioluminescence specifically in response to CAI-1
BB170	*V*. *harveyi* luxN::Tn5	Produces bioluminescence specifically in response to AI-2

### Preparation of autoinducers

Preparation of cloned AIs was conducted using cultures of *E*. *coli* DH5α carrying recombinant plasmids pJZ176 and pJZ365 containing genes for CAI-1 and AI-2 synthase respectively were used to prepare the respective autoinducer. These recombinant plasmids carried the *cqsA* and *luxS* genes cloned in the vector pTAC under a *lac* promoter [4,13]. Overnight culture of each recombinant clone grown in LB containing 50μg/ml of ampicillin was diluted in fresh LB medium containing the same antibiotic and grown further until the OD_600_ reached 0.5–1.0. The culture was centrifuged for 15 min at 4500 x g to precipitate cells, and the cells were resuspended in an original volume of fresh LB medium without any antibiotic, and containing 0.5mM IPTG. After incubation at 37°C with shaking for another 6h, the culture supernatant was collected by centrifugation to precipitate the cells. The supernatant which contained the autoinducer was filtered through 0.22μm pore-sized Millipore filters to sterilize as described previously [4,17].

To prepare AIs from different *V*. *cholerae* strains, the relevant strain was grown overnight for approximately 18h in LB at 37°C with shaking. The culture was centrifuged to precipitate cells and collect the supernatant. Culture supernatant which contained the AIs was sterilized by filtration through 0.22μm pore-sized filters to sterilize.

### Bioluminescence assay for CAI-1 and AI-2 activity

Two bioluminescence reporter strains MM920 and BB170 were used to assay the activity of CAI-1 and AI-2 respectively in cell-free culture supernatants of *V*. *cholerae* or *E*. *coli* strains as described previously [12,19,20]. In brief, the autoinducer activity induced the reporter cells to produce light which was measured and the bioluminescence was expressed as relative light units (RLU). Single colonies of the respective strains were grown in 5ml LB medium overnight at 37°C with shaking. The cultures were diluted in fresh LB and grown to an optical density of 1.5 at 600nm (OD_600_ = 1.5). Cells were precipitated from the cultures by centrifugation, and the supernatants were sterilized by filtration through a 0.22μm pore sized filter. The presence of CAI-1 or AI-2 activity in the cell-free culture supernatants were tested as follows.

Overnight culture of the reporter strain grown in LB medium with shaking at 30°C, was diluted 1:10 in fresh LB, and 70 ml aliquots were transferred to a 96-well opaque-wall microtitre plate. The filter-sterilized culture supernatant of the test strains were added to a final concentration of 30% (v/v), and the plates were incubated for 2.5 to 4h at 30°C with shaking. Culture supernatants prepared in the same way using derivatives of *V*. *cholerae* strain C6706 carrying deletions of the relevant indigenous autoinducer synthase gene and hence unable to produce the autoinducer were included in the reporter assays as negative controls. Subsequently, light production and OD_600_ values were recorded. Bioluminescence was expressed in Relative light units (RLUs) defined as light production (counts per minute) divided by OD_600_ as described previously [9].

### Probes and PCR assays

Presence of defined mutations in the *cqsS* gene carried by *V*. *cholerae* non-O1 non-O139 strains was initially determined by a combination of specific PCR and DNA probe assays. The PCR assay using two primer 5’-TTGTGCGGCAGCGGTGCTGTTC and 5’-TTCACAGCTAACGGTTTTGCAA based on the sequence of the typical *cqsS* gene of strain N16961 [21], was designed to exclude strains carrying a set of defined mutations in the *cqsS* gene. This PCR assay would produce the desired amplicon from typical *V*. *cholerae* strain carrying normal version of the *cqsS* gene but would fail to amplify *cqsS* gene carrying the defined mutations as found in the AI-2 overproducing non-O1 non-O139 strains (S1 and S2 Figs). The probe used in the hybridization assays, was a PCR amplicon of the *cqsS* gene that was radioactively labeled using a random primers DNA labeling kit (Invitrogen Corporation, Carlsbad, CA) and [α-^32^P]-deoxycytidine triphosphate (3,000 Ci/mmol) [22]. Colony blots were prepared using nylon filters (Hybond, Amersham Biosciences, Uppsala, Sweden) and processed by standard methods [23]. The colony blots were hybridized with the labeled probes and autoradiographs were developed as described previously [24].

### DNA sequencing

*V*. *cholerae* genomes were sequenced at the genomics facility of icddr,b using Illumina based technology. For whole genome sequencing, Illumina Nextera^®^ XT DNA library Preparation Kit (Cat. no, FC-131-1024) was used as per manufacturer’s instructions, to prepare Genomic fragment libraries, and sequencing was conducted with an Illumina MiSeq sequencer. The sequences were assembled, and aligned with reference sequences using software available at Illumina BaseSpace (https://basespace.illumina.com/lab) on-line.

### Resuscitation of *V*. *cholerae* O1 CVEC in water samples

Environmental water samples which were initially found negative for *V*. *cholerae* O1 in conventional enrichment culture were subjected to this assay as described previously [17]. In brief, a mixture of 3 ml of the water sample, 3 ml of AI preparation and 3ml of fresh LB medium was incubated at 37°C with shaking. Aliquots of the culture were withdrawn after 3h and 5h of enrichment and plated on TTGA containing an appropriate antibiotic as described previously [7]. Water samples treated in the same way but using culture supernatants of negative control strains or fresh LB instead of the supernatant were incubated and analyzed in parallel, as controls.

### Statistical analysis

General statistical analysis of data was done using the data analysis program built-in Microsoft Excel (MS office version 2007) and were expressed as mean ± standard deviation. The differences were analyzed by two-tailed t-test, and values of p<0.05 were considered significant. Correlation between the increase in prevalence of *V*. *cholerae* O1 and that of the AI-2 overproducing non-O1 non-O139 strains was tested by Pearson’s correlation coefficient and calculating significance using the online calculator at https://www.socscistatistics.com/pvalues/pearsondistribution.aspx.

### GenBank accession numbers

The whole genome shotgun projects for various strains have been deposited at DDBJ/ENA/GenBank under the Accession numbers JADNQP000000000, JADNQQ00000000, and JADNQR000000000, for *V*. *cholerae* non-O1 non-O139 strains 126V0117, 107V1216, and 86V1216 respectively.

### Institutional approvals

Experimental protocols were reviewed and approved by the Research Review Committee, and Ethics Review Committee of the International Centre for Diarrhoeal Disease Research, Bangladesh (Protocol numbers PR-15029 and PR-07018).

## Results

### Identification of *V*. *cholerae* non-O1 non-O139 strains which overproduce AI-2

**Fig 1:**
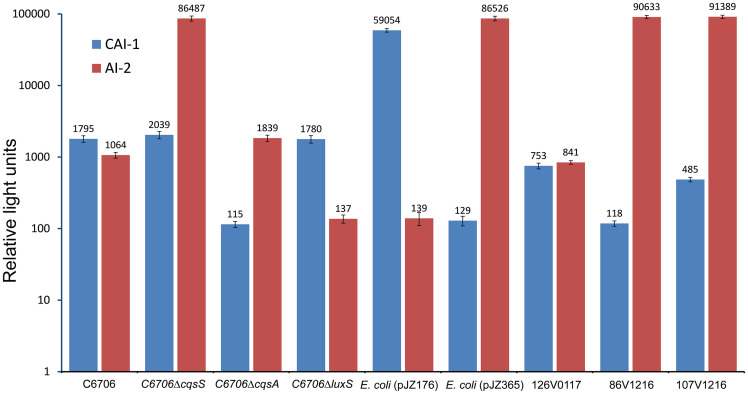
Activity of autoinducers CAI-1 and AI-2 produced by different strains as estimated using bioluminescence reporter assays, and expressed as relative light units (RLU, see text for details). Recombinant *E*. *coli* strains carrying plasmids pJZ176 and pJZ365 produce CAI-1 and AI-2 respectively in their culture supernatants. Strains 126V0117, 86V1216 and 107V1216 are environmental *V*. *cholerae* non-O1 non-O139 isolates. Strain C6706 is a laboratory strain of *V*. *cholerae* O1, C6706Δ*cqsS* carry deletions of the gene encoding the receptor for CAI-1, whereas C6706Δ*cqsA* and C6706Δ*luxS* carry deletions of genes for biosynthesis of CAI-1 and AI-2 respectively.

*V*. *cholerae* produces at least three autoinducers namely CAI-1 and AI-2, and DPO [9], of which the former two have been shown to enhance resuscitation of CVEC, dormant forms of *V*. *cholerae* present in environmental water samples [4]. To identify *V*. *cholerae* non-O1 non-O139 variant strains which might overproduce either of the two autoinducers CAI-1 and AI-2 associated with CVEC resuscitation, we initially analyzed 61 different isolates of *V*. *cholerae* non-O1 non-O139 from water samples using two bioluminescent reporter strains MM920 and BB170 [12,19,20]. The non-O1 non-O139 strains were obtained primarily from water samples that also yielded *V*. *cholerae* O1 in enrichment culture. The two reporter strains MM920 and BB170 expressed bioluminescence specifically in response to autoinducer CAI-1 and AI-2 respectively, and were used to estimate these autoinducers in the culture supernatants of different strains. As control samples, in these assays we included culture supernatant of *E*. *coli* DH5α strains harboring recombinant plasmids carrying genes for the synthesis of CAI-1 or AI-2. For comparison, we also included spent culture supernatants of a typical *V*. *cholerae* O1 strain C6706 and a *V*. *cholerae* non-O1 non-O139 strain 126V0117. As negative controls we also included derivatives of strain C6706 in which the indigenous genes for synthesis of CAI-1 or AI-2 had been deleted [17]. We identified 2 strains of *V*. *cholerae* non-O1 designated as 86V-1216 and 107V-1216, both of which overproduced the autoinducer AI-2 in their culture supernatants (Fig 1). While their CAI-1 production was comparable to that of the control strains C6706 and 126V0117, the level of AI-2 produced by these variant strains was about a 100 fold higher than that produced by the typical strains C6706 or 126V0117. Subsequently, we analyzed possible genetic changes that could have caused over-expression of AI-2 by strains 86V-1216 and 107V-1216 as described later.

### Genomic analysis of *V*. *cholerae* non-O1 non-O139 strains which overproduce AI-2

**Fig 2:**
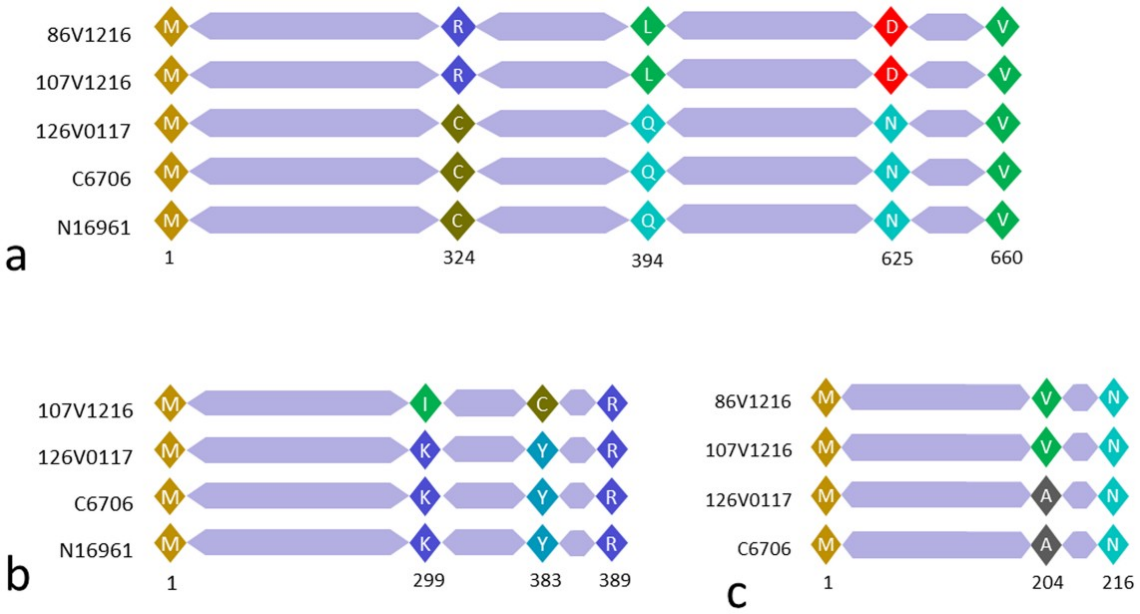
Multiple alignment of predicted amino acid sequences of (a) cqsS (b) cqsA and (c) HapR proteins. Strains 86V1216 and 107V1216 are AI-2 overproducing *V*. *cholerae* non-O1 non-O139 isolated from water samples, whereas strains C6706 and N16961, and 126V0117 are typical *V*. *cholerae* O1 and non-O1 non-O139 respectively. Notably, strain 86V1216 does not carry the *cqsA* gene encoding CAI-1 synthase, and strain N16961 does not have an active *hapR* gene due to a natural frame shift mutation [21].

Three *V*. *cholerae* non-O1 non-O139 strains were subjected to whole genome sequencing. These included two strains 86V-1216 and 107V-1216 which overproduced AI-2, and the control non-O1 non-O139 strain 126V0117 which did not overproduce the autoinducer. The total genomic size of these strains were respectively 4055854bp, 3894935bp and, 3935348bp, and were comparable in size with the genomes of two typical *V*. *cholerae* O1 strains namely N16961 (4033464bp) and C6706 (4019194 bp), found in the GenBank data base. A total of six genes of the quorum sensing pathway (*cqsS*, *cqsA*, *luxS*, *luxO*, *luxU*, *hapR*), and five housekeeping genes encoding 6-phosphofructokinase, glucose-6-phosphate dehydrogenase, citrate synthase, malate dehydrogenase, and NAD kinase, carried by the non-O1 non-O139 strains and the *V*. *cholerae* O1 strains N16961 and C6706 were compared by multiple sequence alignment. The products of the 5 house keeping genes were found to be conserved with no change at the amino acid level. On the other hand, the predicted *cqsS* gene products in the two non-O1 non-O139 strains 86V1216, and 107V1216 were significantly altered in several positions compared to the control O1 and non-O1 non-O139 strains. In particular, a cysteine residue at position 324 of the predicted cqsS protein was replaced by an Arginine residue (Fig 2a). Notably, cysteine carries an uncharged polar side chain but arginine has charged basic side chain. Likewise, the polar amino acid glutamine was replaced by a nonpolar amino acid leucine at position 394 in these two non-O1 non-O139 strains. Also, asparagine at position 625 was replaced by aspartate (Fig 2a). Notably, unlike asparagine aspartate has charged acidic side chain. Most other gene products of the quorum sensing pathway were found to be similar in all strains analyzed, except that the *cqsA* gene encoding CAI-1 synthase was absent in the non-O1 non-O139 strain 86V1216, whereas in strain 107V1216 the predicted protein product of *cqsA* gene had replacement in two amino acids. Precisely, lysine was replaced by leucine at position 299. Whereas Lysine is a charged basic amino acid, leucine is nonpolar. Besides, tyrosine was replaced by cysteine at position 383, and both of them are polar amino acids (Fig 2b). The hapR protein in these two non-O1 non-O139 strains were slightly altered as compared to strain C6706 (Fig 2c). Namely, alanine in position 204 was replaced by valine, and both these residues are nonpolar in nature. Notably, *V*. *cholerae* strain N16961 does not have an active *hapR* gene due to a natural frameshift mutation [21].

### Culture supernatants of non-O1 non-O139 strains carrying mutations in *cqsS* gene enhance resuscitation of CVEC

As culture supernatants of the *V*. *cholerae* non-O1 non-O139 strains which sustained mutations in the *cqsS* gene contained elevated level of AI-2 as shown in the bioluminescence reporter assays, we examined whether the supernatants also caused resuscitation of the dormant *V*. *cholerae* cells or CVEC [4] in water samples obtained from the aquatic environment. The water samples were collected in Dhaka, Bangladesh, from sites where similar water samples were previously found to contain CVEC [4]. Samples were initially tested for the presence of *V*. *cholerae* by enrichment and culture [7], to exclude water samples which might contain culturable *V*. *cholerae* cells at low concentration. Only samples that were negative in the initial enrichment culture were subjected to the resuscitation assays. Supplementation of culture medium by adding 50% vol:vol spent culture medium from the non-O1 non-O139 strains carrying mutant *cqsS* gene was found to cause reactivation of dormant *V*. *cholerae* O1 cells (Table 2). Notably, there was no recovery of cells in the first 3h of incubation, but viable cells appeared at high numbers (10^4^-10^5^cfu/ml) suddenly after 5h of incubation. Culture supernatants of recombinant *E*. *coli* strains expressing either CAI-1 or AI-2, and that of *V*. *cholerae* strain C6706 carrying deletion in its *cqsS* gene were also used in parallel assays, and resuscitation of CVEC also occurred in the presence of these culture supernatants. However, when enriched similarly but using spent medium of *V*. *cholerae* strain C6706 deleted of the AI synthase genes, and that of *E*. *coli* DH5α with the empty vector pTAC, aliquots of these same water samples were mostly found negative for culturable *V*. *cholerae* (Table 2). Similarly, in complementation assays culture supernatants of *cqsS* deleted strains or the non-O1 non O139 strains with mutated *cqsS* gene carrying a plasmid clone of the wild type *cqsS* gene failed to resuscitate CVEC in the water samples.

**Table 2:** Recovery of *Vibrio cholerae* O1 CVEC in water samples by culturing under different conditions.

Date of sampling	No. of samples tested^b^	Number of samples found positive for *V*. *cholerae* O1 following enrichment in medium containing spent medium^a^ of different strains										
		(A) C6706	(B) DH5α (pTAC)^c^	(C) DH5α (pJZ176)^c^	(D) DH5α (pJZ364)^c^	(E) *C6706*Δ*cqsA*Δ*luxS*	(F) *C6706*Δ*cqsS*	(G) *C6706*Δ*cqsS* (pcqsS)	(H) 86V1216	(I) 86V1216 (pcqsS)	(J) 107V1216	(K) 107V1216 (pcqsS)
Aug-16	11	2	1	7	5	0	7	1	8	0	7	1
Sep-16	10	1	0	6	5	1	7	1	6	0	6	2
Oct-16	11	0	0	9	6	0	6	0	7	0	5	1
Nov-16	10	0	1	6	4	0	8	0	8	2	7	0
Dec-16	10	1	0	7	4	1	8	2	6	1	6	0
Jan-17	9	1	0	4	6	0	7	0	6	1	5	1
Feb-17	12	1	1	7	3	0	8	0	5	0	5	0
Mar-17	10	0	0	7	5	0	7	3	7	1	6	2
Apr-17	11	1	0	7	5	0	6	1	5	1	8	2
May-17	12	2	1	9	5	0	4	2	7	1	4	0
Jun-17	12	2	0	7	4	0	6	0	7	1	9	2
Jul-17	14	1	0	6	5	1	6	0	8	0	6	0
Aug-17	10	2	1	5	4	0	9	1	7	1	6	1
Sep-17	12	1	0	8	6	1	8	0	7	0	5	0
Oct-17	11	0	0	6	4	2	6	0	4	0	5	0
Total samples	165	15	5	101	71	6	103	11	98	9	90	12
% of total	100	9.0	3.0	61.2	43.0	3.6	62.4	6.6	59.3	5.4	54.5	7.2

^a^The enrichment medium comprised of LB supplemented with spent medium (50%, v/v) of the indicated strain. Samples were inoculated into the enrichment medium and then incubated for 5 hours at 37°C. Aliquots of enrichment culture were removed after 3h and 5h and plated on TTGA plates containing streptomycin.

^*b*^CVEC positive water samples are shown.

^*c*^*pJZ176* and *pJZ364* are recombinant plasmids carrying *cqsA* and *luxS* genes respectively, whereas *pTAC* is the empty cloning vector.

When compared between columns: B vs C, B vs D, A vs C, A vs D, F vs G, H vs I, and J vs K respectively, the differences in recovery of culturable cells by enrichment under different conditions as described were found to be statistically significant (p <0.001).

### Environmental prevalence of *V*. *cholerae* O1 coincides with that of non-O1 non-O139 strains which overproduce AI-2

We conducted systematic environmental sampling to explore the prevalence of *V*. *cholerae* O1 as well as the variant forms of *V*. *cholerae* non-O1 non-O139 which overproduce AI-2. Representative *V*. *cholerae* isolates obtained from different rounds of sampling were initially analyzed for a defined mutation in their *cqsS* gene using a combination of PCR and DNA probe assays. Sampling was done monthly from August 2016 to November 2017, spanning three cholera seasons in Bangladesh. All water samples were analyzed for *V*. *cholerae* O1 or O139 as well as non-O1 non-O139 strains by an enrichment culture technique [7]. While the presence or absence of *V*. *cholerae* O1 was monitored in water samples, from each water sample, at least 3 non-O1 non-O139 *V*. *cholerae* which failed to agglutinate with O1 and O139 serogroup specific anti-sera were saved for further analysis.

These isolates were initially subjected to a PCR assay for the *cqsS* gene using a set of primers that were designed to deliberately exclude strains carrying the defined mutations in *cqsS* gene. We sought to identify strains which were negative in PCR, although they were positive for the gene in a *cqsS* specific polynucleotide DNA probe assay (S1 Fig). Isolates which were negative for *cqsS* in PCR despite being positive in the DNA probe assay were expected to carry the defined mutations. Accordingly, between 10–90% of the non-O1 non-O139 strains isolated at different sampling periods were negative in the PCR assay but positive in the DNA probe assay, suggesting that the *cqsS* gene in these isolates sustained the defined mutations in the *cqsS* gene (Table 3), and were likely overproducers of AI-2. Subsequently, we tested these isolates to verify their ability to overproduce AI-2 using the reporter strain, and as expected all these isolates were found to overproduce AI-2 by nearly 100 fold compared to control strains (S2 Fig). Remarkably, the frequency of detection of *V*. *cholerae* O1 in environmental water samples increased with a general increase in the concentration of these variant strains of *V*. *cholerae* non-O1 non-O139 in water (Table 3), and the observed Pearson’s correlation coefficient (R = 0.9468) between the increase in prevalence of *V*. *cholerae* O1 and that of the AI-2 overproducing non-O1 non-O139 strains was highly significant (p<0.001).

**Table 3:** Co-occurrence of *Vibrio cholerae* non-O1 non-O139 strains carrying defined mutations in the *cqsS* gene and overproducing AI-2 with toxigenic *V*. *cholerae* O1 in surface water samples in Bangladesh^d^.

Month and Year	Number (%) of water samples^a^ positive for *V*. *cholerae* O1	Number of *V*. *cholerae* non-O1 non-O139 isolates analyzed^b^	Number (%) of isolates carrying mutations in the *cqsS* gene (PCR negative and probe positive)^c^
Aug-16	3 (15)	72	11 (15.2)
Sep-16	5 (25)	80	18 (22.5)
Oct-16	15 (75)	91	75 (82.4)
Nov-16	14 (70)	90	75 (83.3)
Dec-16	2 (10)	75	25 (33.3)
Jan-17	1 (5)	62	10 (16.1)
Feb-17	4(20)	65	21 (32.3)
Mar-17	12(60)	83	75 (90.3)
Apr-17	17(85)	91	80 (87.9)
May-17	15(75)	86	72 (83.7)
Jun-17	1(5)	77	8 (10.3)
Jul-17	3(15)	75	5 (6.6)
Aug-17	2(10)	87	12 (13.7)
Sep-17	10(50)	72	60 (83.3)
Oct-17	15 (75)	95	79 (83.1)
Nov-17	14 (70%)	82	62 (75.6)

^a^In each round of sampling a total of 20 water samples from 10 sampling sites were collected and analyzed for *V*. *cholerae* O1.

^b^Isolates were randomly picked from environmental water samples which were subjected to standard enrichment and culture.

^c^Initial screening of the isolates was done by PCR and DNA probe assays for the *cqsS* gene. Isolates which were negative in PCR but positive in probe assays, were found to overproduce AI-2 as assayed using the bioluminescent reporter strain.

The correlation between increase in the number of samples positive for *V*. *cholerae* O1 and the abundance of the *V*. *cholerae* non-O1 non-O139 strains carrying a mutant cqsS gene (R = 0.9468) was statistically significant (p<0.001).

## Discussion

The results of this study provide several new insights into the environmental biology of the cholera pathogen toxigenic *V*. *cholerae* O1 and its ecological relation to the highly ubiquitous non-O1 non-O139 *V*. *cholerae*. These results might help to explain the observed increased abundance of the pathogen in its aquatic reservoirs periodically which presumably enhances transmission leading to seasonal cholera epidemics. Quorum sensing autoinducers (AIs) of *V*. *cholerae* such as CAI-1 and AI-2 have been shown to act as resuscitation factors for CVEC, the dormant environmental form of *V*. *cholerae* O1 found in water samples, when assayed in the laboratory [4]. We have previously shown that certain *V*. *cholerae* O1 mutant strains may overproduce AI-2 which resuscitate dormant toxigenic *V*. *cholerae* in such water samples [17] under laboratory conditions. However, evidence for a role of such mutant strains in resuscitation of CVEC under natural conditions remains inadequate. Since the vast majority of naturally occurring *V*. *cholerae* belong to serogroups other than O1 or O139, collectively known as *V*. *cholerae* non-O1 non-O139, presence and periodic blooms of probable AI-overproducing non-O1 non-O139 strains would significantly influence the resuscitation process. Therefore, in this study we sought to identify and genetically characterize *V*. *cholerae* non-O1 non-O139 strains which overproduce one or more AIs (Fig 1). We found that strains which were identified as overproducers of AI-2 carry certain defined mutations in the *cqsS* gene (Fig 2). Notably, mutational inactivation of the same gene, was also found to cause overproduction of AI-2 by *V*. *cholerae* O1 strains in a previous study [17].

Each of the AI-2 overproducing non-O1 non-O139 strains analyzed in the present study, was found to have sustained a number of mutations in the nucleotide sequence of the *cqsS* gene (Fig 2a) resulting in three substitutions of amino acids in the predicted cqsS protein. Among these, a cysteine residue at position 324 was replaced by an Arginine residue. Cysteine is an important amino acid known for its function in tertiary and quaternary structure formation of proteins through forming disulfide bonds. Therefore, absence of this amino acid is likely to have affected the conformation of the protein and presumably rendered it inactive. However, further studies are necessary to confirm this assumption through appropriate laboratory experiments to demonstrate that the altered protein is indeed unable to bind with the autoinducer CAI-1. Likewise, the polar amino acid glutamine was replaced by a nonpolar amino acid leucine at position 394 in these two non-O1 non-O139 strains. Also, asparagine at position 625 was replaced by aspartate (Fig 2a). Unlike asparagine, aspartate has a charged acidic side chain. We confirmed using specific bioluminescence assays, that these *cqsS* mutant strains indeed overproduced AI-2 (Fig 1). Moreover, the AI-2 produced in the culture supernatants of these strains were functional and could enhance resuscitation of dormant *V*. *cholerae* O1 present in environmental water samples (Table 2).

Although the precise mechanism involved in the overproduction of AI-2 by these strains remains unclear, our observations agree with a previous study [17] demonstrating that all AI-2 overproducers carry mutations in the *cqsS* gene encoding the CAI-1 receptor. We presume that inability to sense CAI-1 by strains with nonfunctional CAI-1 receptor leads to trigger an alternative pathway that boost production of AI-2. However, extensive further studies including defined mutagenesis and functional complementation would be required to work out the precise pathway that lead to this observed hyper-production of AI-2 by both *V*. *cholerae* O1 strains as shown previously [17], and by the non-O1 non-O139 strains. Nevertheless, in the present study we focused our investigation on possible influence of such AI-2 overproducing variant strains on natural resuscitation of CVEC, which is likely to be a crucial step in the initiation of seasonal cholera epidemics in endemic areas [5].

To begin to assess the possibility that under natural conditions, a subpopulation of *V*. *cholerae* non-O1 non-O139 strains which overproduce AI-2 might contribute to resuscitation of dormant toxigenic *V*. *cholerae*, we monitored the correlation between increased occurrence of toxigenic *V*. *cholerae* O1 with that of *V*. *cholerae* non-O1 non-O139 carrying certain defined mutations in the *cqsS* gene and overproducing AI-2, in water samples collected from aquatic sites in Bangladesh. Sampling for this study was done monthly from August 2016 to November 2017, spanning three seasonal epidemics of cholera. All water samples were analyzed for *V*. *cholerae* O1 or O139 as well as non-O1 non-O139 strains by standard enrichment culture techniques. Representative isolates were subjected to further genetic analysis.

A combination of PCR and DNA probe assays, as well as bioluminescence assays with a reporter strain were used in this analysis. In our initial screening, about 10–90% of the non-O1 non-O139 strains isolated at different sampling periods and 0.1% of environmental *V*. *cholerae* O1 isolates tested were negative in a PCR assay for the *cqsS* gene but were positive for the gene in the DNA probe assay (Table 3). The PCR primers were designed to exclude strains that carried defined mutations in the cqsS gene as found in the AI-2 overproducing strains. To further confirm this assumption, we conducted bioluminescence assay for AI-2 using the reporter strain and found these to be indeed AI-2 hyper producing strains.

The environmental prevalence of *V*. *cholerae* non-O1 non-O139 strains carrying mutations in the c*qsS* gene and overproducing AI-2 was found to fluctuate (Table 3). When the proportion of such variant strains increased in the aquatic environment compared to typical non-O1 non-O139 strains, the frequency of isolation of *V*. *cholerae* O1 also increased. This positive correlation expressed as Pearson’s Correlation Coefficient (R = 0.9468) was statistically significant (p<0.001). Besides, any water sample that was positive for *V*. *cholerae* O1 also yielded a high proportion (>5 out of 10 isolates tested) of *V*. *cholerae* non-O1 non-O139 isolates which overproduced AI-2.

In the Ganges Delta regions of Bangladesh and India, cholera epidemics occur in a seasonal pattern with usually two epidemics each year [1,2], and dormant forms of pathogenic *V*. *cholerae* O1 that exist in aquatic reservoirs periodically resuscitate prior to occurrence of seasonal epidemics [3–6]. The resuscitation can be enhanced in the laboratory by exposure to either of two AI types, CAI-1 and AI-2 [4]. We presume that the resuscitation of dormant strains referred to as CVEC could occur in aquatic niches through exposure to AI-2 molecules produced by *V*. *cholerae* non-O1 non-O139, which are abundant in natural waters, and have genetic variants that overproduce AI-2.

Of 3 different autoinducers produced by *V*. *cholerae* [9], CAI-1 is used for communication within the Vibrio genus, whereas AI-2 and DPO are used for communication among diverse species of bacteria [10,12,25]. Since, certain autoinducers in particular AI-2 is produced by numerous bacterial species [26–28], the contribution of species other than *V*. *cholerae* in resuscitation of CVEC can not be ruled out. The resuscitation of CVEC could occur either in the aquatic reservoir due to AI-2 produced by diverse bacterial species or inside the host through possible exposure to AI molecules produced by the normal intestinal microbiota present in humans ingesting *V*. *cholerae*. However, the observed correlation of *V*. *cholerae* O1 prevalence with that of non-O1 strains overproducing AI-2 in the environment suggest significant contribution of the non-O1 non-O139 *V*. *cholerae*. It may be mentioned that in our previous studies [4,17] as well as in the present study most typical *V*. *cholerae* strains were found to produce autoinducers at a relatively lower concentration compared to the AI-2 overproducing mutant strains (Fig 1 and S2 Fig), and was not sufficient to resuscitate CVEC in laboratory assays.

If seasonal changes in environmental parameters such as temperature, salinity, carbon content, and the presence of other aquatic organisms give rise to environmental blooms of organisms that produce AI-2 or such *V*. *cholerae* non-O1 non-O139 strains that over produce AI-2, then nearby dormant *V*. *cholerae* O1 might resuscitate and more readily increase in number and cause human disease during this period. This concept could explain aspects of cholera seasonality in areas such as the Ganges Delta, where Bangladesh is located. Although, resuscitation of CVEC might also occur inside the human gut through the influence of AI molecules produced by normal gastrointestinal microbiota, the role of environmental organism which seasonally fluctuate in concentration can perhaps better explain cholera seasonality.

Other pathogenic bacteria, such as *Vibrio vulnificus*, *Shigella sonnei*, *Shigella flexneri*, *Campylobacter jejuni*, *E*. *coli*, *Salmonella enteritidis*, and *Legionella pneumophila*, have also been shown to convert to a dormant state after exposure to environmental stresses [5,29,30]. If autoinducers play a role in the resuscitation of dormant cells of other species as well, these results may have implications in understanding the epidemiology and transmission of bacterial disease beyond cholera as such. Consequently, the possibility that seasonal occurrence of cholera might depend on bacterial communication through autoinducer signals, may lead to further molecular ecological studies as novel means for surveillance, prevention and control of waterborne bacterial infections.

## Supporting information

S1 Fig Combination of of PCR and DNA probe assays to identify *V*. *cholerae* strains carrying a defined mutation in their *cqsS* gene. Primers were designed to exclude strains carrying the defined mutation from amplification of the *cqsS* gene. Subsequently all isolates were subjected to colony blot hybridization using a *cqsS* gene probe to identify PCR negative but probe positive strains. A gel showing (a) PCR results and (b) an autoradiograph derived from colony blot hybridization assays is shown.
(DOCX)

S2 Fig Activity of autoinducers produced by different strains and their derivatives as assayed using bioluminescence produced by reporter strains for CAI-1 and AI-2, expressed as relative light units (RLU, see text for details). Recombinant plasmid pJZ365 carries the gene for AI-2 synthase required for biosynthesis of AI-2. Strains VCO3 and VCO9 are environmental *V*. *cholerae* O1 strains. Strains VCN23 and VCN27 are environmental non-O1 non-O139 strains which were positive in the PCR assay for the cqsS gene, whereas strains designated as VCN42, VCN54, VCN56, VCN72, VCN87, and VNC 29 were V. cholerae non-O1 non-O139 strains which were negative in the PCR assay but positive in the DNA probe assay for *cqsS* gene. C6706 is a laboratory strain of *V*. *cholerae* O1.
(DOCX)
