# Supplementary material for: Environmental prevalence of toxigenic Vibrio cholerae O1 in Bangladesh coincides with V. cholerae non-O1 non-O139 genetic variants which overproduce autoinducer-2
Source: PLoS One. 2021 Jul 2;16(7):e0254068. doi: 10.1371/journal.pone.0254068 (PMC8253391; doi:10.1371/journal.pone.0254068)
Supplement: S1 Fig — Primers were designed to exclude strains carrying the defined mutation from amplification of the cqsS gene. Subsequently all isolates were subjected to colony blot hybridization using a cqsS gene probe to identify PCR negative but probe positive strains. A gel showing (a) PCR results and (b) an autoradiograph derived from colony blot hybridization assays is shown. (DOCX) [file pone.0254068.s001.docx]

**
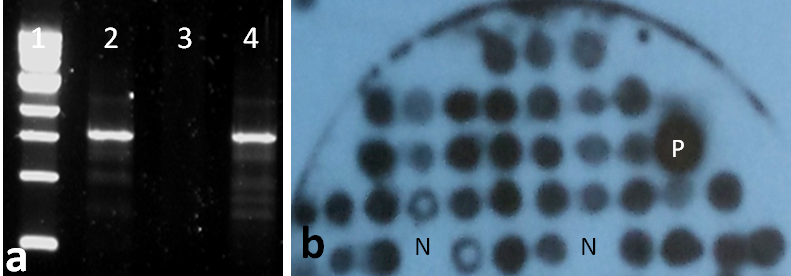
**

**Figure S1.** Combination of of PCR and DNA probe assays to identify *V. cholerae* strains carrying a defined mutation in their *cqsS* gene. Primers were designed to exclude strains carrying the defined mutation from amplification of the cqsS gene. Subsequently all isolates were subjected to colony blot hybridization using a cqsS gene probe to identify PCR negative but probe positive strains. A gel showing (a) PCR results and (b) an autoradiograph derived from colony blot hybridization assays is shown.
