# Supplementary material for: Environmental prevalence of toxigenic Vibrio cholerae O1 in Bangladesh coincides with V. cholerae non-O1 non-O139 genetic variants which overproduce autoinducer-2
Source: PLoS One. 2021 Jul 2;16(7):e0254068. doi: 10.1371/journal.pone.0254068 (PMC8253391; doi:10.1371/journal.pone.0254068)
Supplement: S2 Fig — Recombinant plasmid pJZ365 carries the gene for AI-2 synthase required for biosynthesis of AI-2. Strains VCO3 and VCO9 are environmental V. cholerae O1 strains. Strains VCN23 and VCN27 are environmental non-O1 non-O139 strains which were positive in the PCR assay for the cqsS gene, whereas strains designated as VCN42, VCN54, VCN56, VCN72, VCN87, and VNC 29 were V. cholerae non-O1 non-O139 strains which were negative in the PCR assay but positive in the DNA probe assay for cqsS gene. C6706 is a laboratory strain of V. cholerae O1. (DOCX) [file pone.0254068.s002.docx]

**
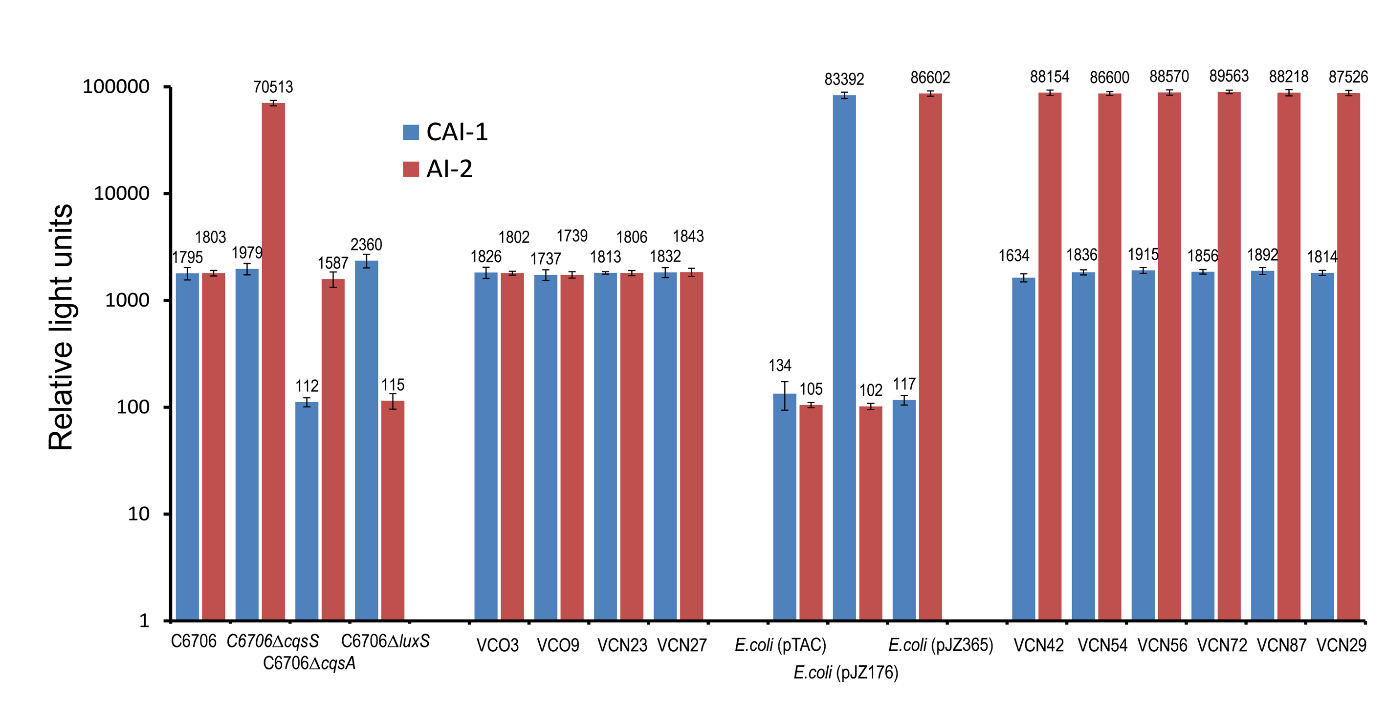
**

**Figure S2**. Activity of autoinducers produced by different strains and their derivatives as assayed using bioluminescence produced by reporter strains for CAI-1 and AI-2, expressed as relative light units (RLU, see text for details). Recombinant plasmid pJZ365 carries the gene for AI-2 synthase required for biosynthesis of AI-2. Strains VCO3 and VCO9 are environmental *V. cholerae* O1 strains. Strains VCN23 and VCN27 are environmental non-O1 non-O139 strains which were positive in the PCR assay for the *cqsS* gene, whereas strains designated as VCN42, VCN54, VCN56, VCN72, VCN87, and VNC 29 were *V. cholerae* non-O1 non-O139 strains which were negative in the PCR assay but positive in the DNA probe assay for cqsS gene. C6706 is a laboratory strain of *V. cholerae* O1.
